# Role of gasdermins in chronic kidney disease

**DOI:** 10.3389/fimmu.2025.1557707

**Published:** 2025-04-01

**Authors:** Hanchao Gao, Ting Xie, Yunyi Li, Zigan Xu, Zhuoheng Song, Huixia Yu, Hongming Zhou, Weilong Li, Chen Yun, Baozhang Guan, Shaodong Luan, Lianghong Yin

**Affiliations:** ^1^ Department of Nephrology, Shenzhen Longhua District Central Hospital, Shenzhen Longhua District Key Laboratory for Diagnosis and Treatment of Chronic Kidney Disease, Shenzhen, Guangdong, China; ^2^ Department of Nephrology, The First Affiliated Hospital of Jinan University, Guangzhou, Guangdong, China; ^3^ Charité-Universitätsmedizin Berlin, Campus Mitte, Berlin, Germany

**Keywords:** gasdermins, pyroptosis, chronic kidney disease, inflammation, renal fibrosis

## Abstract

Gasdermins (GSDMs), functioning as membrane perforating proteins, can be activated by canonical inflammasomes, noncanonical inflammasomes, as well as non-inflammasomes, leading to cell pyroptosis and the subsequent release of inflammatory mediators. Increasing evidence has implicated that GSDMs are associated with chronic kidney disease (CKD), including diabetes nephropathy, lupus nephritis, obstructive nephropathy, and crystalline nephropathy. This review centers on the role of GSDMs-mediated pyroptosis in the pathogenesis of CKD, providing novel ideas for enhancing the prognosis and therapeutic strategies of CKD.

## 1 Introduction

Chronic kidney disease (CKD), a pressing concern in global public health, imposes a considerable disease burden and poses a major challenge (1). CKD ranked as the 16th most common cause of death globally in 2016 and is expected to rise to 5th by 2040 (2). The global prevalence of CKD is about 9.1%, while awareness of CKD among the general population and high-risk groups, at just only 6% and 10% (3, 4). Currently, the definition of CKD is structural destruction or impaired function of the kidney lasting 3 months or more (4). Renal fibrosis, a common pathological manifestation of CKD, is characterized by glomerulosclerosis, tubular atrophy, interstitial fibrosis, and persistent inflammation (5, 6). With the continuous destruction of renal tissue, renal function progressively declines, and eventually it will develop into end stage renal disease. The ensuing need for renal replacement therapies, including peritoneal dialysis, hemodialysis, and kidney transplantation, undoubtedly further exacerbate the economic burden on patients.

Pyroptosis, a necrotic programmed cell death mediated by gasdermins (GSDMs), is characterized by cell membrane rupture and the release of inflammatory factors such as interleukin-1 (IL-1β) and interleukin-18 (IL-18) (7, 8). GSDMs were initially identified in macrophages in 1992 and subsequently named in 2001 (9, 10). Shao et al. characterized GSDMs as the executors of pyroptosis, and the in 2018, the definition was revised to cell death involving GSDMs-formed membrane pores (7, 11, 12). Pyroptosis primarily occurs in myeloid phagocytes, including macrophages, dendritic cells, and neutrophils, with evidence later confirming its presence in CD4^+^ T cells, keratinocytes, epithelial cells, and neurons as well (13, 14). Pyroptosis is involved in innate immunity by eliminating pathogens, including bacteria, fungi, and viruses, through the mediation of inflammatory response. However, uncontrolled pyroptosis has the potential to induce inflammation in adjacent cells and tissues, thereby contributing to the initiation and progression of diseases (15–17).

Recently, a wealth of evidence indicates that GSDMs-mediated pyroptosis plays a pivotal role in the pathogenesis and progression of CKD. This paper presents an overview of the fundamental characteristics of the GSDMs family and examines and summarizes their roles and potential mechanisms in CKD, providing novel insights for enhancing the prognosis and treatment of this condition.

## 2 Gasdermins

Gasdermins were initially discovered in the gastrointestinal tract and skin of mice, hence they were named “Gasdermins (GSDMs)” (18, 19). In humans, there are six GSDMs genes: *GSDMA, GSDMB, GSDMC, GSDMD, GSDME* (also known as *DNFA5*), and *PJVK* (pejvakin, also known as *GSDMF, DFNB59*). In mice, there are three *Gsdma* genes *(Gsdma*1-3), four *Gsdmc* genes (*Gsdmc*1-4), *Gsdmd*, *Gsdme*, and *Pjvk*, while the *Gsdmb* orthologs are lacking (20, 21) (**Table 1**). In kidney tissue, GSDMD was highly expressed, GSDMC and GSDME were moderately expressed, GSDMB was low, and GSDMA was not detected (21). Except for PJVK, members of the GSDMs protein family are composed of three parts, namely, the N-terminal pore-forming domain (PFD, NTD), the linker part, and the C-terminal inhibitory domain (RD, CTD) (22). When the body is exposed to various internal and external stimuli, GSDMs undergo cleavage by cysteinyl aspartate-specific protease (Caspases) or granzymes, and the dissociated N-terminal structural oligomers punch holes in the cell membrane, mediating the release of inflammatory factors, as well as the flux of water and electrolyte flow, ultimately leading to pyroptosis.

**Table 1:** Expression and activator of gasdermins.

Gene	Chromosome location		Main expression sites	Activator
	Human	Mice		
*GSDMA*	17q21	11D	skin, upper gastrointestinal tract	SpeB Caspase-3/4/7
*GSDMB*	17q21	–	gastrointestinal tract, thyroid, skin, lungs and kidney	GZMA Caspase-1/3/4/6/7
*GSDMC*	8q24.1	15	skin, spleen, trachea, gastrointestinal tract	Caspase-6/8
*GSDMD*	8q24.3	15	sophagus, stomach, skin	Caspase-1/4/5/11
*GSDME*	7p15	6B2.3	gastrointestinal tract, kidney	Caspase-3
*PJVK*	2q31.2	2C3	auditory pathway	–

### 2.1 GSDMA

Human *GSDMA* is located on chromosome 17 and is mainly expressed in epithelial cells of the skin and upper digestive tract (18). *Gsdma1* is expressed specifically in the heart region, *Gsdma2* is expressed in the fundus and pylorus, and *Gsdma3* is mainly expressed in the skin (19). Overexpression of GSDMA is associated with glioma immune escape and poor prognosis in patients, while GSDMA knockdown increases T cell antitumor response via immunotherapy (23). GSDMA can promote the development of esophageal cancer and cisplatin resistance (24). In addition, GSDMA has been implicated in systemic sclerosis, asthma, intestinal bowel disease (IBD), and other immune-related diseases (25–27). However, the role of GSDMA in kidney disease has not been previously reported.

### 2.2 GSDMB

Human *GSDMB* is also located on chromosome 17 and is mainly expressed in the gastrointestinal tract, thyroid, skin, lungs, and kidneys (18, 28). Besides normal tissues, GSDMB is also highly expressed in tumor tissues, such as bladder cancer, clear cell renal cell carcinoma, gastric cancer, and non-small cell lung cancer (29–32). Furthermore, GSDMB is also associated with IBD, asthma, allergic rhinitis, psoriasis, and other diseases (33–36).

### 2.3 GSDMC

Human *GSDMC* is located on chromosome 8 (8q24.1), while mouse *Gsdmc* is located on chromosome 15, and is mainly expressed in skin, spleen, trachea, and gastrointestinal tract (37–39). Under hypoxic conditions, GSDMC transcription was enhanced in breast tumor cells, and GSDMC in turn converted TNF-α-induced apoptosis to pyroptosis (40). In addition, GSDMC is also associated with esophageal cancer, liver cancer, colorectal cancer, pancreatic cancer, and renal clear cell carcinoma (41–46).

### 2.4 GSDMD

Similar to *GSDMC*, human *GSDMD* is also located on chromosome 8 (8q24.3), and mouse *Gsdmd* is located on chromosome 15, and is predominantly expressed in tissues such as the gastrointestinal tract, kidney, and skin (47, 48). GSDMD is the first member of the GSDM family to be found to perform pyroptosis, and the mechanism is well understood (49). GSDMD is mainly cleaved by Caspases at the Asp275 or Asp276 site, Caspase-1/4/5 in humans and Caspase-1/11 in mice (47, 50). GSDMD is involved in a variety of inflammatory diseases such as non-alcoholic steatohepatitis, acute pancreatitis, colitis, sepsis, systemic lupus erythematosus, psoriasis (51–55), and is also associated with acute myocardial infarction, stroke, colon cancer, and other non-inflammatory diseases (56–58). In addition, GSDMD is associated with renal disease, which is described in detail below.

### 2.5 GSDME

*GSDME*, also known as deafness, autosomal dominant 5 (*DFNA5*), is localized on chromosome 7 in humans and chromosome 6 in mice, exhibiting ubiquitous expression across various normal tissues, notably including the gastrointestinal tract and kidney (59). In addition to tumors, GSDME is also involved in hearing loss, neurodegeneration, atherosclerosis, and many renal diseases, which are described in detail below (60–64).

### 2.6 PJVK

*PJVK*, situated on chromosome 2 in both humans and mice, is majored expresses in the inner ear and involved in auditory pathways (65). Mutations in *PJVK* can lead to hearing loss (66). The role of PJVK in renal diseases has not been described.

## 3 Molecular mechanism of pyroptosis

**Figure 1:**
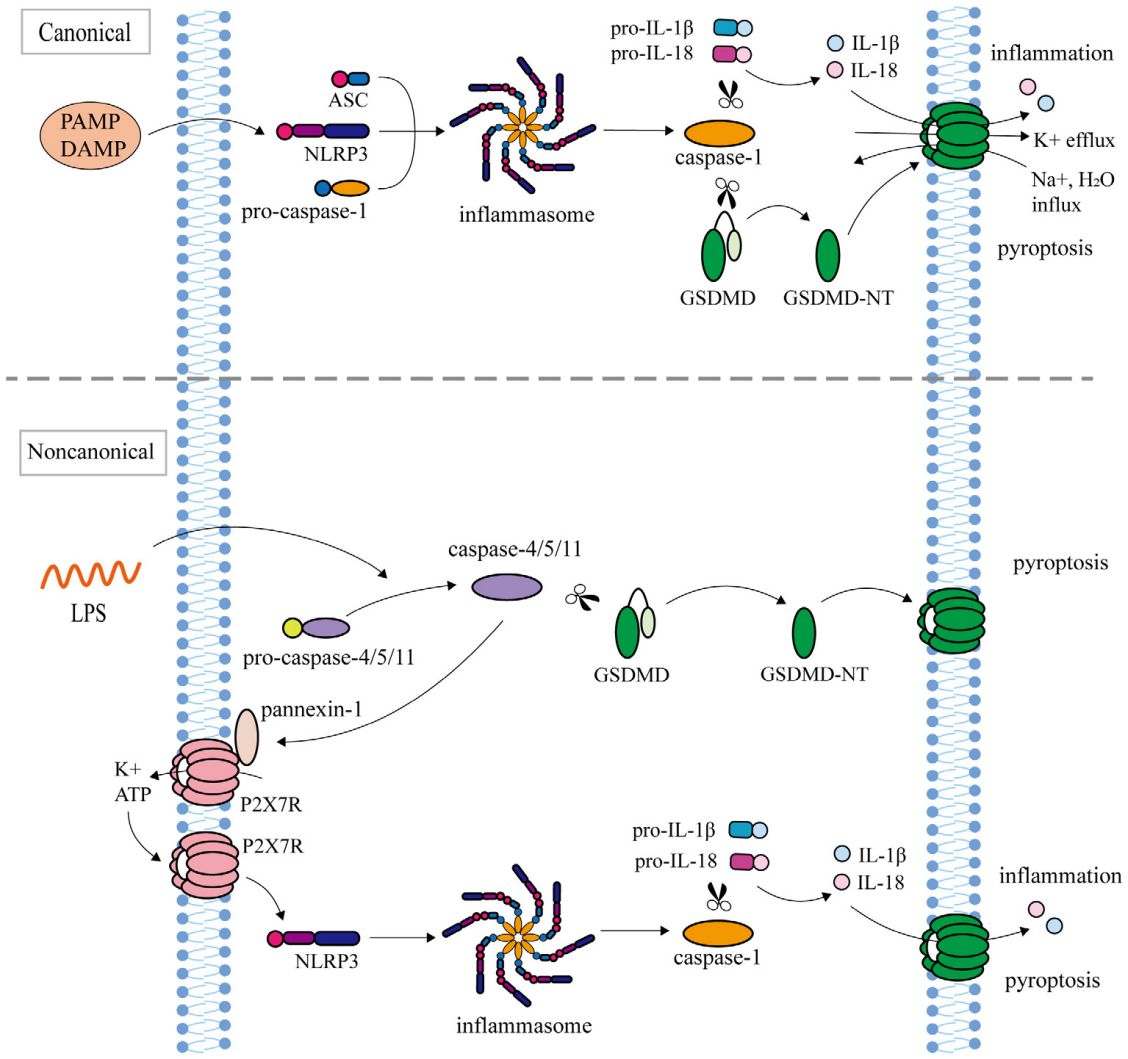
Inflammasome-dependent pathway (canonical pathway and non-canonical pathway). The canonical pathway: NLRP3, ASC, and pro-Caspase-1 assemble into inflammasome in response to PAMP or DAMP. After inflammasome assembly, activated Caspase-1 cleaves pro-IL-1β and pro-IL-18 to mature IL-1β and IL-18, and simultaneously cleaves GSDMD to release the N terminus of GSDMD. GSDMD-NT oligomerize in the cell membrane to form transmembrane pores, causing K^+^ efflux, Na^+^ and water influx, followed by cell swelling and rupture, releasing IL-1β and IL-18, leading to pyroptosis. The non-canonical pathway: inflammasome Caspase-4/5/11 recognizes Lipopolysaccharide (LPS). Activated Caspase-4/5/11 cleaved GSDMD to induce pyroptosis. Caspase-11 activates the Pannexin-1 channel and releases ATP, which further activates the NLRP3 inflammasome to indirectly cut pro-IL-1β and pro-IL-18.

**Figure 2:**
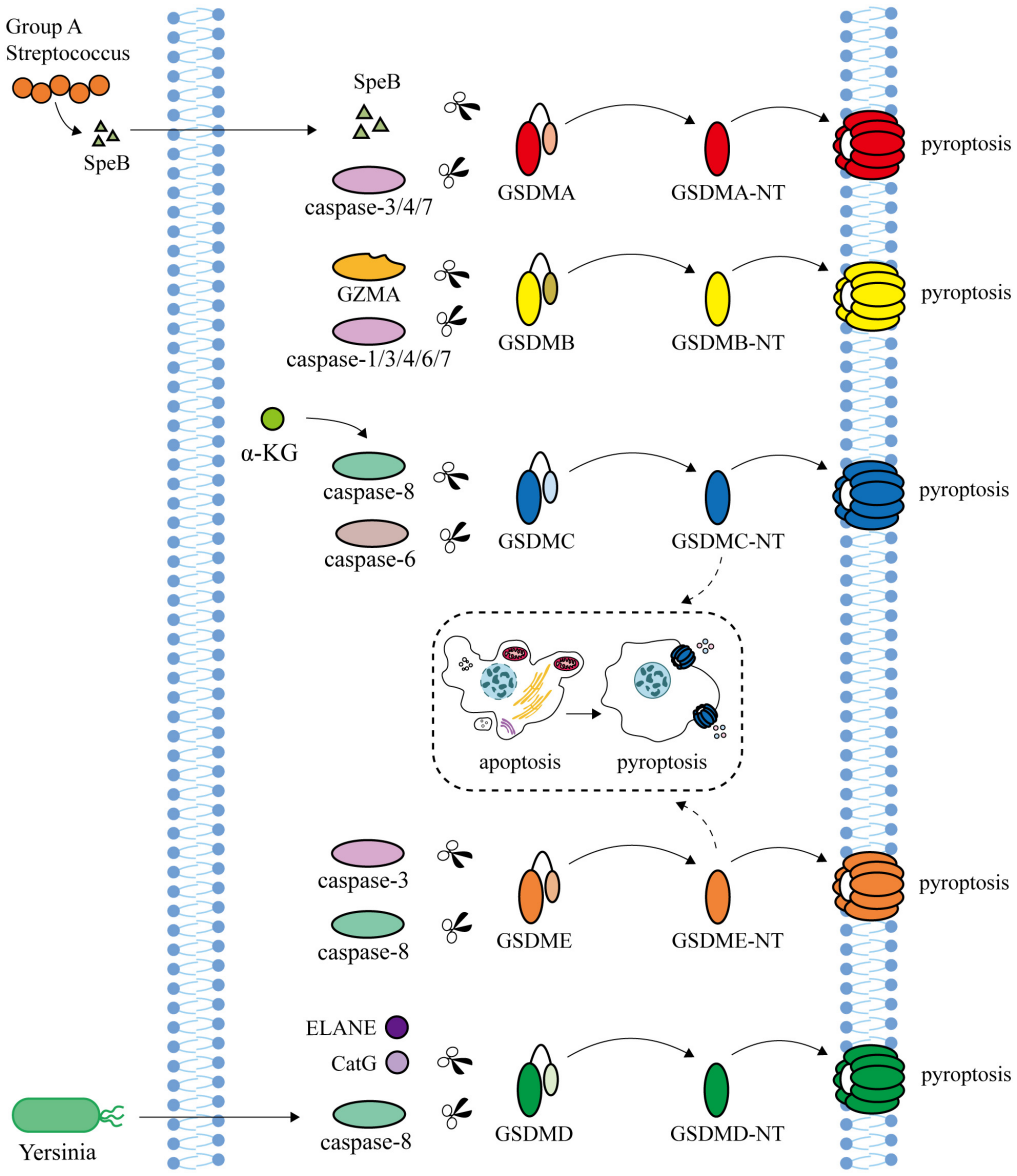
Non-inflammasome-dependent pathway. Streptococcal exotoxin B (SpeB), secreted by human pathogen Group A Streptococcus (GAS), and Caspase-3/4/7 are able to cleave GSDMA to induce pyroptosis. Granzyme A (GZMA), secreted by natural killer cells and cytotoxic T lymphocytes, and Caspase-1/3/4/6/7 cleave GSDMB. α-ketoglutarate (α-KG) induces pyroptosis through Caspase-8 cleavage at GSDMC. In addition, GSDMC is also cleaved by Caspase-6 to induce pyroptosis. Neutrophil elastase (ELANE) and cathepsin G (CatG) can activate GSDMD. Caspase-8 activated by Yersinia can cleave GSDMD and GSDME to trigger pyroptosis. GSDMC and GSDME can transform apoptosis into pyroptosis.

At present, there are two main types of pyroptosis pathways mediated by Gasdermins: inflammasome-dependent pathway (canonical pathway and non-canonical pathway) and non-inflammasome-dependent pathway (**Figures 1**, **2**).

### 3.1 Canonical inflammasome-dependent pathway

In the canonical pathway, inflammasomes are intricate multi-molecular assemblies that encompass pattern recognition receptors (PRRs), apoptosis-associated speck like protein (ASC), and pro-Caspase-1 (67). As sensors that receive pathogen-associated molecular patterns (PAMP) or damage associated molecular patterns (DAMP) stimuli, PRRs mainly include NLR/NOD-like receptors (NLRP1, NLRP3, NLRP6, NLRP9, NLRC4), Pyrin, and AIM2-like receptor (68). NLRP1 can be activated by Anthrax lethal toxin (LeTx), bacterial muramyl dipeptide, T. gondii, and ATP depletion (69). NLRP3 senses a variety of stimuli, including microbial toxins, viral RNA, ATP, ROS, uric acid crystals, cholesterol, and particulate matter (70–72). Microbial RNA, metabolites, Lipoteichoic acid, and LPS can be used as NLRP6 activation signals (73–75). NLRP9 and AIM2 inflammasomes can recognize pathogen dsDNA (76, 77). Bacterial flagellin can induce the activation of NLRC4 inflammasome (78). Pyrin cannot directly recognize PAMP or DAMP but can be activated when bacterial toxins induce GTPase inactivation (79). PAMP or DAMP activates TLRs/MyD88/NF-κB signaling pathway, leading to transcriptional upregulation of NLRP3, ASC, pro-Caspase-1, pro-IL-1β, and pro-IL-18 (80, 81). After inflammasome assembly, activated Caspase-1 cleaves pro-IL-1β and pro-IL-18 to mature IL-1β and IL-18, and simultaneously cleaves GSDMD to release the N terminus of GSDMD (7, 82). GSDMD-NTs oligomerize in the cell membrane to form transmembrane pores to release IL-1β and IL-18 as well as lead to pyroptosis (15, 83).

### 3.2 Non-canonical inflammasome-dependent pathway

In the non-canonical pathway, inflammasomes, human Caspase-4/5 and mouse Caspase-11, specifically recognize the lipopolysaccharide (LPS) by N-terminal CARD (84). The cleavage of GSDMD by Caspase-4/5/11 triggers the process of pyroptosis (47, 50). Caspase-11 fails to cleave pro-IL-1β and pro-IL-18 like Caspase-4/5, but instead activates Pannexin-1 and releases ATP. Subsequently, ATP binds to the P2X7 receptor (P2X7R), causing K^+^ efflux and further activating NLRP3 inflammasome to indirectly cleave pro-IL-1β and pro-IL-18 (85).

### 3.3 Non-inflammasome-dependent pathway

The human pathogen Group A Streptococcus (GAS) secretes the cysteine protein streptococcal exotoxin B (SpeB), which cleaves GSDMA at the junction area of Gln246 and triggers pyroptosis of skin epithelial cells and keratinocytes, independent of Caspase (86, 87). In HEK293T cells, Caspase-3/4/7 are able to cleave GSDMA to induce pyroptosis (88). Granzyme A (GZMA), secreted by natural killer cells and cytotoxic T lymphocytes, cleaves GSDMB at the K244 site of the junction and targets phospholipids on the membrane of Gram-negative bacteria to directly kill bacteria (89, 90). GSDMB can bind to Caspase-1/3/4/6/7 and promote pyroptosis (91, 92). It has also been reported that activated Caspase-7 cuts GSDMB at the D91 site to block GSDMB-mediated pyroptosis (93). α-Ketoglutarate (α-KG) contributes to pyroptosis by enabling Caspase-8 to cleave GSDMC at the Asp240 site, with a similar cleavage occurring at the Asp233 site in mouse GSDMC4 (94). In addition, GSDMC is also cleaved by Caspase-6 to induce pyroptosis (40). Under hypoxia conditions, the increased expression of GSDMC can transform the apoptosis induced by Caspase-8 into pyroptosis (95). Besides Caspase-1/4/5/11, neutrophil elastase (ELANE) and cathepsin G (CatG) can also activate GSDMD (96, 97). Caspase-8 activated by Yersinia can cleave GSDMD and GSDME to trigger pyroptosis (98). GSDME can also be specifically cleaved by Caspase-3 at the Asp270 site to induce pyroptosis, which is transformed into apoptosis after GSDME knockout or promoter demethylation (11, 95, 99, 100).

## 4 Gasdermins and CKD

**Figure 3:**
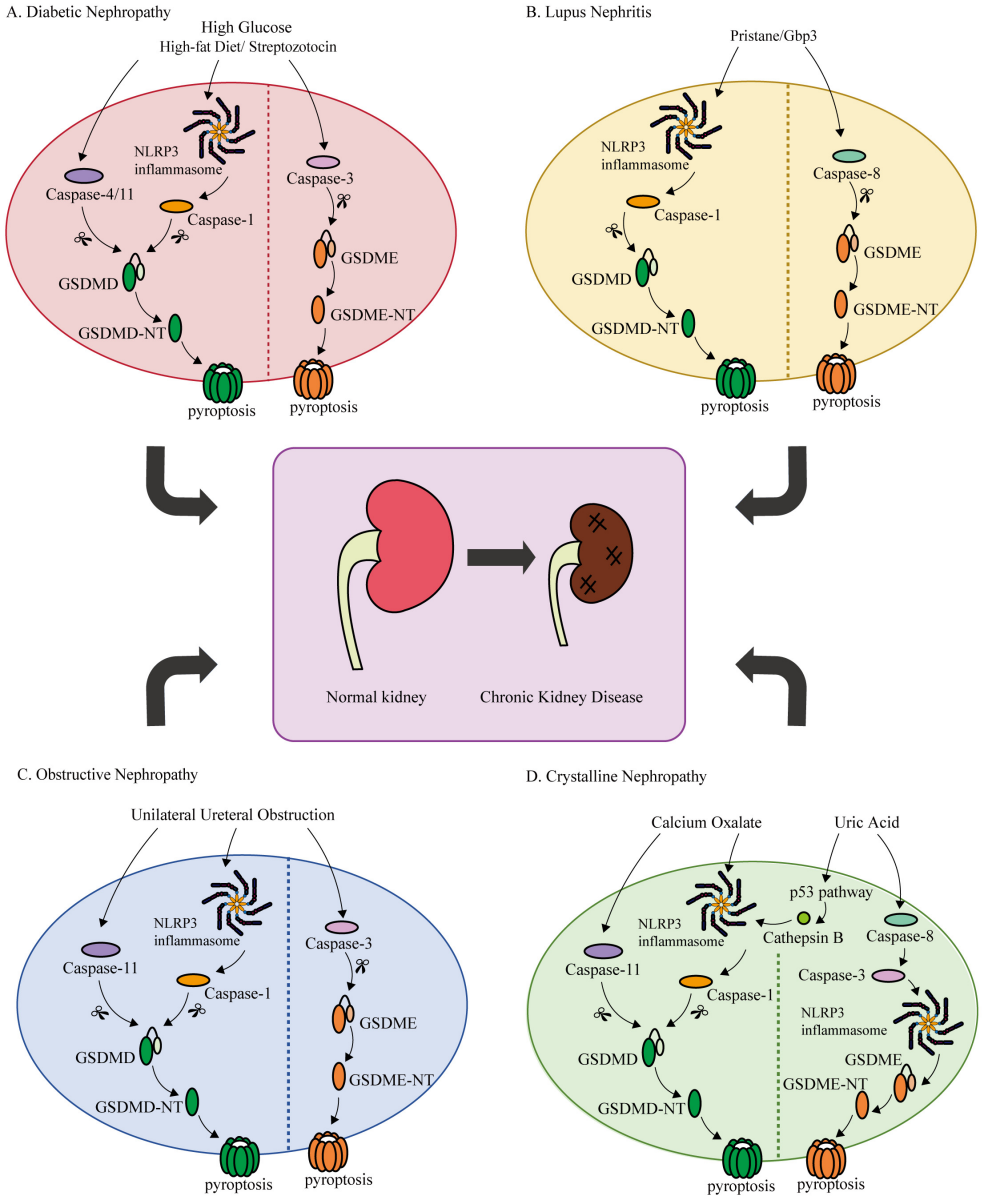
Summary of potential mechanisms of GSDMD/GSDME-mediated pyroptosis in chronic kidney disease. **(A)** Diabetic Nephropathy. Under high glucose or high-fat diet/streptozotocin stimulation, GSDMD is activated by Caspase-1/4/11, while GSDME is activated by Caspase-3, triggering pyroptosis, leading to impaired renal function, increased inflammation and fibrosis. **(B)** Lupus Nephritis. After the generation of autoantibodies by injection of pristane, GSDMD and GSDME can be cleaved by Caspase-1 and Caspase-8, respectively. Subsequently, pyroptosis and renal damage occur. **(C)** Obstructive Nephropathy. Following obstruction of the ureter, pyroptosis induced by Caspase-1/11/GSDMD and Caspase3/GSDME pathways can be activated, mediating renal injury, inflammation, and fibrosis. **(D)** Crystalline Nephropathy. Calcium oxalate can induce pyroptosis and cause renal damage through the Caspase-1/11/GSDMD pathway. Uric acid releases cathepsin B through the p53 pathway and subsequently activates the Caspase-1/11/GSDMD pathway. Furthermore, uric acid directly triggered Caspase-8/Caspase-3/NLRP3/GSDME-mediated pyroptosis, resulting in kidney damage.

Inflammation and fibrosis are prevalent pathological characteristics in all forms of CKD, and persistent inflammation can drive fibrosis progression, leading to the pathogenesis of CKD (5, 101). Damaged cells secrete pro-inflammatory cytokines to recruit inflammatory cells for infiltration, and release pro-fibrotic mediators to promote proliferation of myofibroblasts, resulting in the production and deposition of a large amount of extracellular matrix, ultimately causing pathological changes and impaired renal function (101, 102). Recently, a great number of studies has demonstrated that GSDMs-mediate pyroptosis, a pro-inflammatory form of cell death, which is associated with the pathogenesis of various CKD, including diabetic nephropathy, lupus nephritis, obstructive nephropathy, and crystal nephropathy (**Table 2**, **Figure 3**).

**Table 2:** Involved gasdermins and roles of gasdermins in chronic kidney disease.

Diseases	Research objects	Stimulation	Gasdermins	Roles	Reference
DN	Podocytes, MGECs, HGECs C*asp-11* ^−/−^ mice, *Gsdmd* ^−/−^ mice, db/db mice	HG HFD/STZ -	GSDMD	Renal functional deterioration, inflammation, glomerular sclerosis, and renal interstitial fibrosis	(105–107)
	HK-2 cells C57BL/6 mice	HG HFD/STZ	GSDMD	Tubular injury, interstitial inflammation, and renal fibrosis	(108)
	HK-2 cells, rat HBZY-1 cells Rats	HG STZ	GSDME	Tubular injury and inflammation	(109)
	DN patients	–	GSDMD	Renal tubulointerstitial fibrosis and renal functional deterioration	(108, 110)
LN	HGECs Podocytes from MRL/lpr mice BALB/c mice MRL/lpr mice, LN patients	LPS LPS+ATP Pristane -	GSDMD	Inflammation	(117, 118)
	HK-2 cells G*sdme* ^−/−^ mice, SLE patients, lupus mice	TNF-α+CHX Pristane -	GSDME	Tubular injury, interstitial inflammation, and renal fibrosis	(119)
ON	*Gsdme* ^−/−^ mice	UUO	GSDME	Inflammation and renal fibrosis	(122)
	RTECs HK-2 cells *Gsdme* ^−/−^ mice	OGSD+TNF-α TGF-β1 5/6 nephrectomy/UUO	GSDME	Tubular injury, inflammation, and renal fibrosis	(63, 123)
CN	HK-2 cells *Gsdmd* ^−/−^ mice	CaOx crystals/Oxalate Glyoxylic acid	GSDMD	Inflammation	(131, 132)
	NRK-52E cells, RTECs *Gsdmd* ^−/−^ mice, HN rats	Uric acid; Potassium oxonate+adenine	GSDMD	Inflammation and renal fibrosis	(138–140)

DN, diabetic nephropathy; MGECs, mouse glomerular endothelial cells; HGECs, human glomerular endothelial cells; HG, high clucose; HFD, high-fat diet; STZ, streptozotocin; HK-2 cells, human kidney proximal tubular epithelial cells; HBZY-1 cells, glomerular mesangial cells; LN, Lupus Nephritis; MRL/lpr mice, MRL/mpj-Fas^lpr^ (MRL/lpr) mice; TNF-α, tumor necrosis factor-α; CHX, cycloheximide; SLE, Systemic Lupus Erythematosus; UUO, unilateral ureteral obstruction; RTECs, renal tubular epithelial cells; OGSD, oxygen-glucose-serum deprivation; TGF-β1, transforming growth factor-beta 1; NRK-52E cells, renal tubular epithelial cells; HN, hyperuricemic nephropathy.

### 4.1 Diabetic nephropathy

Diabetic nephropathy (DN) is a prevalent microvascular complication of diabetes mellitus, emerging as a major type of CKD (103). Pathological changes of diabetic nephropathy include cells damage (podocytes, renal tubular epithelial cells (RTECs), endothelial cells), glomerular sclerosis, glomerular basement membrane thickening, mesangial matrix increase, tubular interstitial inflammation, and fibrosis (104).

Recent studies have revealed a correlation between GSDMs-mediated pyroptosis and DN, suggesting a potential mechanistic link between the two processes. In a recent study, high glucose (HG) or streptozotocin (STZ) stimulated pyroptosis in renal cells (podocytes, renal tubules, human glomerular endothelial cells, and mouse glomerular endothelial cells) though the Caspase-11/GSDMD pathway, resulting in impaired renal function, manifested by significant increases in serum nitrogen, serum creatinine, and urinary albumin levels (105). After knocking down the *Gsdmd* gene in DN mice, pyroptosis and IL-18 were markedly reduced, resulting in improved renal function (105).An et al. showed that punicalagin effectively inhibit NLRP3/Caspase-1/GSDMD/IL-1β pathway, and significantly ameliorates renal function, glomerular sclerosis, renal interstitial fibrosis and other pathological changes in diabetic mice (106). Cheng et al. also demonstrated that the expression levels of Caspase-4/11, GSDMD-N, IL-1β, and IL-18 were markedly elevated in HG-treated podocytes or podocytes from diabetic mice, and the deficiency of *Caspase-11* or *Gsdmd* alleviated inflammation and podocytes loss in diabetic mice (107). Immunohistochemistry staining showed that the levels of fibronectin, alpha-smooth muscle actin, and the number of F4/80^+^ macrophages in DN mice were higher than the control group (108). Western blotting results indicated that the expressions of GSDMD-FL, GSDMD-NT, IL-1β, IL-18, and α-SMA were increased in DN mice, suggesting that pyroptosis was associated with renal tubule injury and interstitial inflammation (108). In the rat DN model and HG-treated human kidney proximal tubular epithelial cells (HK-2 cells), Caspase-1/3, GSDME, GSDME-NT, and IL-1β were significantly upregulated. In rats that underwent renal cortical injection of AAV9-shGSDME to knockdown *Gsdme* in the kidney, reduced renal injury and pyroptosis were observed, demonstrating that Caspase-3/GSDME promotes pyroptosis and contributes to renal injury in DN (109).

In addition, pyroptosis was also detected in renal biopsy tissues obtained from DN patients. When compared with patients with glomerular minor lesion, the expressions of Caspase-1 and GSDMD in renal tubular cells were increased in DN patients (108). Increased expression of GSDMD in DN patients was positively correlated with renal tubulointerstitial fibrosis and negatively correlated with renal function (110). Additionally, the expression of NLRP3 was upregulated in DN patients, and demonstrating a positive association with tubulointerstitial injury (111).

These studies indicate that GSDMD and GSDME are activated in DN, and GSDMD or GSMDE mediated-pyroptosis promotes the pathogenesis of DN.

### 4.2 Lupus nephritis

Systemic lupus erythematosus (SLE) is a multisystem autoimmune disease characterized by autoantibody formation, immune complex deposition, and chronic inflammation (112). Kidneys are the affected organs in SLE, with up to 60% of patients developing lupus nephritis (LN) (113). The pathological mechanism of LN involves the deposition of immune complexes, infiltration of inflammatory cells, and injury or death of glomerular and tubular cell (114).

Studies have shown that characteristic autoantibodies and typical clinical and renal pathological changes of SLE can be produced after intraperitoneal injection of pristane (115, 116). Increased expressions of pyroptosis related proteins (NLRP3, GSDMD, and Caspase-1) and inflammatory factors (TNF-α and IL-1β) were observed in pristane-induced LN mice models (117). Cao et al. treated LN patients and MRL/lpr mice with a combination of mycophenolate mortiate, calcineurin inhibitors, and steroids, resulting in significant inhibition of Caspase-1/GSDMD-mediated pyroptosis and alleviation of disease progression (118). GSDME was observed to be highly expressed in the renal tubules of SLE patients and pristane-induced lupus mice, while Caspase-3/GSDME-mediated pyroptosis, autoantibody, glomerular IgG deposition and renal lesions were ameliorated in *Gsdme* ^-/-^ lupus mice (119).

### 4.3 Obstructive nephropathy

The causes of obstructive nephropathy (ON) include urinary calculi, congenital stenosis, and tumor masses. The main characteristics of ON include renal dysfunction, inflammation, and renal interstitial fibrosis, which may progress to CKD with the progression of the disease (120, 121).

Several studies have used the unilateral ureteral obstruction (UUO) model to investigate the role of GSDMs in ON. In UUO model, Caspase-11 was activated to cleave GSDMD to mediate pyroptosis, which induced the formation neutrophil extracellular trap and the transformation of macrophage to myofibroblast, promoting inflammation and renal fibrosis (122). Furthermore, Li et al.’s research suggested that Caspase3/GSDME-mediated pyroptosis of renal tubular cells promoted the progression of renal injury and fibrosis induced by ureteral obstruction (63). In the UUO mice model, inhibition of *Caspase-3* or loss of *Gsdme* was found to alleviate renal fibrosis (123). Disulfiram has been reported to directly inhibit GSDMD cleavage, thereby suppressing pyroptosis and cell fibrosis; however, it is ineffective in inhibiting pyroptosis once the process has already been initiated (124). Furthermore, Tongluo Yishen Detection has been proven to effectively inhibit pyroptosis and improve renal function and fibrosis by targeting NLRP3/Caspase-1/GSDMD pathway (125). YiShen HuoXue decoction can also inhibit this pathway to improve renal fibrosis and inflammation (126).

### 4.4 Crystalline nephropathy

Crystals, including calcium oxalate (CaOx) and uric acid (UA), that deposit in the renal tubules and interstitium induce ischemia, tubule fibrosis, and inflammation through mechanisms involving tubular obstruction and cytotoxicity, ultimately leading to crystalline nephropathy (CN) (127, 128).

Oxalate nephropathy, although rare, has a poor prognosis and often develops into the final stage of CKD (129, 130). Studies have shown that CaOx induced RTECs and HK-2 cells injury through NLRP3/Caspase-1/GSDMD pathway (131, 132). H3 relaxin, with its anti-inflammatory and antioxidant properties, exerted a protective effect against oxalate nephropathy in rats by targeting the NLRP3/Caspase-1/GSDMD axis (133). Melatonin alleviated RTECs damage and renal CaOx crystal deposition by inhibiting non-canocial inflammasome mediated pyroptosis by NLRP3/Caspase-11/GSDMD (134).

Recent studies have shown that hyperuricemia plays a pathogenic role in the progression of kidney disease, leading to CKD (hyperuricemic nephropathy, HN) (135–137). GSDMD was significantly upregulated in renal tissue of experimental HN mice, and the loss of renal function and renal tubule fibrosis were improved after *Gsdmd* knockout (138). A study has shown that UA activated autophagy through the p53 pathway and released cathepsin B, which subsequently activated the NLRP3/Caspase-1,11/GSDMD pathway to participate in the development of experimental HN (139). A. manihot L. flower mitigates UA-induced RTECs damage by inhibiting Caspase-8/Caspase-3/NLRP3/GSDME-mediated pyroptosis (140).

Current studies have shown that GSDMs-mediated pyroptosis is involved in experimental CN, but its role in human CN remains to be determined.

### 4.5 Other experimental CKD

Animal models of CKD provide powerful experimental basis for further exploring the pathogenesis of CKD, verifying the effectiveness of treatment methods. 5/6 nephrectomy (5/6Nx), by removing one whole kidney and two-thirds of the opposite kidney, simulates human CKD manifestations including renal fibrosis and decreased kidney function (141, 142). It is shown that *Gsdme* deletion significantly improves renal function and renal interstitial fibrosis in mice underwent 5/6Nx (123). Adenine-induced renal injury is also commonly used to mimic human CKD. Data indicated that pyroptosis-related proteins such as GSDMD were upregulated in adenine-induced CKD mice, and pyroptosis was significantly alleviated after butyrate intervention (143).

## 5 Conclusion

Here, we provide an overview indicating that GSDMD/GSDME-mediated pyroptosis is an important factor in the pathogenesis of CKD. However, several issues remain to be explored and elucidated through further research. First, it has been established that GSDMD and GSDME are associated with multiple types of CKD; however, the role of GSDMDA, GSDMB, GSDMC, and PJVK in CKD remains unclear. Second, in addition to being activated by Caspases to trigger pyroptosis, GSDMs can convert apoptosis to pyroptosis in some cases, so whether there is mutual conversion or interaction between pyroptosis and apoptosis in CKD. Third, at present, in animal and cellular experiments, some drugs and knocking out/down GSDMs can improve CKD; however, clinical trials are needed to verify. Therefore, we hope that future studies will focus on the design and development of drugs that specifically target GSDMD/GSDME, with the aim of improving the quality of life of CKD patients and will also further explore the functional roles by other members of GSDMs.

## References

[1] Ke C Liang J Liu M Liu S Wang C. Burden of chronic kidney disease and its risk-attributable burden in 137 low-and middle-income countries, 1990-2019: results from the global burden of disease study 2019. BMC Nephrol. (2022) . 23:17. doi: 10.1186/s12882-021-02597-3 34986789 PMC8727977

[2] Foreman KJ Marquez N Dolgert A Fukutaki K Fullman N McGaughey M. Forecasting life expectancy, years of life lost, and all-cause and cause-specific mortality for 250 causes of death: reference and alternative scenarios for 2016-40 for 195 countries and territories. Lancet. (2018) . 392:2052–90. doi: 10.1016/S0140-6736(18)31694-5 PMC6227505 30340847

[3] Collaboration, G.B.D.C.K.D. Global, regional, and national burden of chronic kidney disease, 1990-2017: a systematic analysis for the Global Burden of Disease Study 2017. Lancet. (2020) . 395:709–33. doi: 10.1016/S0140-6736(20)30045-3 PMC7049905 32061315

[4] Kidney Disease: Improving Global Outcomes, C.K.D.W.G. KDIGO 2024 clinical practice guideline for the evaluation and management of chronic kidney disease. Kidney Int. (2024) . 105:S117–314. doi: 10.1016/j.kint.2023.10.018 38490803

[5] Liu Y. Cellular and molecular mechanisms of renal fibrosis. Nat Rev Nephrol. (2011) . 7:684–96. doi: 10.1038/nrneph.2011.149 PMC4520424 22009250

[6] Webster AC Nagler EV Morton RL Masson P. Chronic kidney disease. Lancet. (2017) . 389:1238–52. doi: 10.1016/S0140-6736(16)32064-5 27887750

[7] Shi J Zhao Y Wang K Shi X Wang Y Huang H. Cleavage of GSDMD by inflammatory caspases determines pyroptotic cell death. Nature. (2015) . 526:660–5. doi: 10.1038/nature15514 26375003

[8] Shi J Gao W Shao F. Pyroptosis: gasdermin-mediated programmed necrotic cell death. Trends Biochem Sci. (2017) . 42:245–54. doi: 10.1016/j.tibs.2016.10.004 27932073

[9] Zychlinsky A Prevost MC Sansonetti PJ. Shigella flexneri induces apoptosis in infected macrophages. Nature. (1992) . 358:167–9. doi: 10.1038/358167a0 1614548

[10] Cookson BT Brennan MA. Pro-inflammatory programmed cell death. Trends Microbiol. (2001) . 9:113–4. doi: 10.1016/s0966-842x(00)01936-3 11303500

[11] Wang Y Gao W Shi X Ding J Liu W He H. Chemotherapy drugs induce pyroptosis through caspase-3 cleavage of a gasdermin. Nature. (2017) . 547:99–103. doi: 10.1038/nature22393 28459430

[12] Galluzzi L Vitale I Aaronson SA Abrams JM Adam D Agostinis P. Molecular mechanisms of cell death: recommendations of the Nomenclature Committee on Cell Death 2018. Cell Death Differ. (2018) . 25:486–541. doi: 10.1038/s41418-017-0012-4 29362479 PMC5864239

[13] Vande WL Lamkanfi M. Pyroptosis. Curr Biol. (2016) . 26:R568–72. doi: 10.1016/j.cub.2016.02.019 27404251

[14] Zhang KJ Wu Q Jiang SM Ding L Liu CX Xu M. Pyroptosis: A new frontier in kidney diseases. Oxid Med Cell Longev. (2021) . 2021:6686617. doi: 10.1155/2021/6686617 34007404 PMC8102120

[15] Kovacs SB Miao EA. Gasdermins: effectors of pyroptosis. Trends Cell Biol. (2017) . 27:673–84. doi: 10.1016/j.tcb.2017.05.005 PMC5565696 28619472

[16] Kesavardhana S Malireddi RKS Kanneganti TD. Caspases in cell death, inflammation, and pyroptosis. Annu Rev Immunol. (2020) . 38:567–95. doi: 10.1146/annurev-immunol-073119-095439 PMC7190443 32017655

[17] Zheng X Chen W Gong F Chen Y Chen E. The role and mechanism of pyroptosis and potential therapeutic targets in sepsis: A review. Front Immunol. (2021) . 12:711939. doi: 10.3389/fimmu.2021.711939 34305952 PMC8293747

[18] Saeki N Kuwahara Y Sasaki H Satoh H Shiroishi T. Gasdermin (Gsdm) localizing to mouse Chromosome 11 is predominantly expressed in upper gastrointestinal tract but significantly suppressed in human gastric cancer cells. Mamm Genome. (2000) . 11:718–24. doi: 10.1007/s003350010138 10967128

[19] Tamura M Tanaka S Fujii T Aoki A Komiyama H Ezawa K. Members of a novel gene family, Gsdm, are expressed exclusively in the epithelium of the skin and gastrointestinal tract in a highly tissue-specific manner. Genomics. (2007) . 89:618–29. doi: 10.1016/j.ygeno.2007.01.003 17350798

[20] Broz P Pelegrin P Shao F. The gasdermins, a protein family executing cell death and inflammation. Nat Rev Immunol. (2020) . 20:143–57. doi: 10.1038/s41577-019-0228-2 31690840

[21] De Schutter E Roelandt R Riquet FB Van Camp G Wullaert A Vandenabeele P. Punching holes in cellular membranes: biology and evolution of gasdermins. Trends Cell Biol. (2021) . 31:500–13. doi: 10.1016/j.tcb.2021.03.004 33771452

[22] Ding J Wang K Liu W She Y Sun Q Shi J. Pore-forming activity and structural autoinhibition of the gasdermin family. Nature. (2016) . 535:111–6. doi: 10.1038/nature18590 27281216

[23] Zhang R Song Q Lin X Du B Geng D Gao D. GSDMA at the crossroads between pyroptosis and tumor immune evasion in glioma. Biochem Biophys Res Commun. (2023) . 686:149181. doi: 10.1016/j.bbrc.2023.149181 37924669

[24] Wang H Wang H Chen J Liu P Xiao X. Overexpressed FAM111B degrades GSDMA to promote esophageal cancer tumorigenesis and cisplatin resistance. Cell Oncol (Dordr). (2024) . 47:343–59. doi: 10.1007/s13402-023-00871-0 PMC12974030 37672204

[25] Terao C Kawaguchi T Dieude P Varga J Kuwana M Hudson M. Transethnic meta-analysis identifies GSDMA and PRDM1 as susceptibility genes to systemic sclerosis. Ann Rheum Dis. (2017) . 76:1150–8. doi: 10.1136/annrheumdis-2016-210645 PMC6733404 28314753

[26] Yu J Kang MJ Kim BJ Kwon JW Song YH Choi WA. Polymorphisms in GSDMA and GSDMB are associated with asthma susceptibility, atopy and BHR. Pediatr Pulmonol. (2011) . 46:701–8. doi: 10.1002/ppul.21424 21337730

[27] Soderman J Berglind L Almer S. Gene expression-genotype analysis implicates GSDMA, GSDMB, and LRRC3C as contributors to inflammatory bowel disease susceptibility. BioMed Res Int. (2015) . 2015:834805. doi: 10.1155/2015/834805 26484354 PMC4592899

[28] Hu Y Wang B Li S Yang S. Pyroptosis, and its role in central nervous system disease. J Mol Biol. (2022) . 434:167379. doi: 10.1016/j.jmb.2021.167379 34838808

[29] He H Yi L Zhang B Yan B Xiao M Ren J. USP24-GSDMB complex promotes bladder cancer proliferation via activation of the STAT3 pathway. Int J Biol Sci. (2021) . 17:2417–29. doi: 10.7150/ijbs.54442 PMC8315027 34326684

[30] Cui Y Zhou Z Chai Y Zhang Y. Upregulated GSDMB in clear cell renal cell carcinoma is associated with immune infiltrates and poor prognosis. J Immunol Res. (2021) . 2021:7753553. doi: 10.1155/2021/7753553 34957313 PMC8702340

[31] Saeki N Komatsuzaki R Chiwaki F Yanagihara K Sasaki H. A GSDMB enhancer-driven HSV thymidine kinase-expressing vector for controlling occult peritoneal dissemination of gastric cancer cells. BMC Cancer. (2015) . 15:439. doi: 10.1186/s12885-015-1436-1 26016667 PMC4446855

[32] Zhang X Liu R. Pyroptosis-related genes GSDMB, GSDMC, and AIM2 polymorphisms are associated with risk of non-small cell lung cancer in a Chinese Han population. Front Genet. (2023) . 14:1212465. doi: 10.3389/fgene.2023.1212465 37359371 PMC10287965

[33] Rana N Privitera G Kondolf HC Bulek K Lechuga S De Salvo C. GSDMB is increased in IBD and regulates epithelial restitution/repair independent of pyroptosis. Cell. (2022) . 185:283–298.e17. doi: 10.1016/j.cell.2021.12.024 35021065 PMC8879997

[34] Li X Christenson SA Modena B Li H Busse WW Castro M. Genetic analyses identify GSDMB associated with asthma severity, exacerbations, and antiviral pathways. J Allergy Clin Immunol. (2021) . 147:894–909. doi: 10.1016/j.jaci.2020.07.030 32795586 PMC7876167

[35] Shamsi BH Chen H Yang X Liu M Liu Y. Association between polymorphisms of the GSDMB gene and allergic rhinitis risk in the Chinese population: a case-control study. J Asthma. (2023) . 60:1751–60. doi: 10.1080/02770903.2023.2185893 36847643

[36] Ji X Chen H Xie L Chen S Huang S Tan Q. The study of GSDMB in pathogenesis of psoriasis vulgaris. PloS One. (2023) . 18:e0279908. doi: 10.1371/journal.pone.0279908 36607980 PMC9821418

[37] Watabe K Ito A Asada H Endo Y Kobayashi T Nakamoto K. Structure, expression and chromosome mapping of MLZE, a novel gene which is preferentially expressed in metastatic melanoma cells. Jpn J Cancer Res. (2001) . 92:140–51. doi: 10.1111/j.1349-7006.2001.tb01076.x PMC5926699 11223543

[38] Wu C Orozco C Boyer J Leglise M Goodale J Batalov S. BioGPS: an extensible and customizable portal for querying and organizing gene annotation resources. Genome Biol. (2009) . 10:R130. doi: 10.1186/gb-2009-10-11-r130 19919682 PMC3091323

[39] Zheng Z Deng W Lou X Bai Y Wang J Zeng H. Gasdermins: pore-forming activities and beyond. Acta Biochim Biophys Sin (Shanghai). (2020) . 52:467–74. doi: 10.1093/abbs/gmaa016 32294153

[40] Hou J Zhao R Xia W Chang CW You Y Hsu JM. PD-L1-mediated gasdermin C expression switches apoptosis to pyroptosis in cancer cells and facilitates tumour necrosis. Nat Cell Biol. (2020) . 22:1264–75. doi: 10.1038/s41556-020-0575-z PMC7653546 32929201

[41] Li M Jiang Q Liu X Han L Chen S Xue R. The pyroptosis-related signature composed of GSDMC predicts prognosis and contributes to growth and metastasis of hepatocellular carcinoma. Front Biosci (Landmark Ed). (2023) . 28:235. doi: 10.31083/j.fbl2810235 37919059

[42] Yan C Niu Y Li F Zhao W Ma L. System analysis based on the pyroptosis-related genes identifies GSDMC as a novel therapy target for pancreatic adenocarcinoma. J Transl Med. (2022) . 20:455. doi: 10.1186/s12967-022-03632-z 36199146 PMC9533512

[43] Xia XH Yin WJ Mao JF Liu P Qin CD Hu JJ. The expression profile of Gasdermin C-related genes predicts the prognosis and immunotherapy response of pancreatic adenocarcinoma. Am J Cancer Res. (2023) . 13:1240–58. PMC10164816 37168356

[44] Cui YQ Meng F Zhan WL Dai ZT Liao X. High expression of GSDMC is associated with poor survival in kidney clear cell cancer. BioMed Res Int. (2021) . 2021:5282894. doi: 10.1155/2021/5282894 34778452 PMC8589493

[45] Tang D Zheng Y Wang G Sheng C Liu Z Wang B. HPV18 E6 inhibits alpha-ketoglutarate-induced pyroptosis of esophageal squamous cell carcinoma cells via the P53/MDH1/ROS/GSDMC pathway. FEBS Open Bio. (2023) . 13:1522–35. doi: 10.1002/2211-5463.13666 PMC10392054 37402485

[46] Miguchi M Hinoi T Shimomura M Adachi T Saito Y Niitsu H. Gasdermin C is upregulated by inactivation of transforming growth factor beta receptor type II in the presence of mutated apc, promoting colorectal cancer proliferation. PloS One. (2016) . 11:e0166422. doi: 10.1371/journal.pone.0166422 27835699 PMC5105946

[47] Burdette BE Esparza AN Zhu H Wang S. Gasdermin D in pyroptosis. Acta Pharm Sin B. (2021) . 11:2768–82. doi: 10.1016/j.apsb.2021.02.006 PMC8463274 34589396

[48] Saeki N Usui T Aoyagi K Kim DH Sato M Mabuchi T. Distinctive expression and function of four GSDM family genes (GSDMA-D) in normal and Malignant upper gastrointestinal epithelium. Genes Chromosomes Cancer. (2009) . 48:261–71. doi: 10.1002/gcc.20636 19051310

[49] Kayagaki N Stowe IB Lee BL O’Rourke K Anderson K Warming S. Caspase-11 cleaves gasdermin D for non-canonical inflammasome signalling. Nature. (2015) . 526:666–71. doi: 10.1038/nature15541 26375259

[50] Devant P Kagan JC. Molecular mechanisms of gasdermin D pore-forming activity. Nat Immunol. (2023) . 24:1064–75. doi: 10.1038/s41590-023-01526-w PMC12379974 37277654

[51] Xu B Jiang M Chu Y Wang W Chen D Li X. Gasdermin D plays a key role as a pyroptosis executor of non-alcoholic steatohepatitis in humans and mice. J Hepatol. (2018) . 68:773–82. doi: 10.1016/j.jhep.2017.11.040 29273476

[52] Silva CMS Wanderley CWS Veras FP Sonego F Nascimento DC Goncalves AV. Gasdermin D inhibition prevents multiple organ dysfunction during sepsis by blocking NET formation. Blood. (2021) . 138:2702–13. doi: 10.1182/blood.2021011525 PMC8703366 34407544

[53] Miao N Wang Z Wang Q Xie H Yang N Wang Y. Oxidized mitochondrial DNA induces gasdermin D oligomerization in systemic lupus erythematosus. Nat Commun. (2023) . 14:872. doi: 10.1038/s41467-023-36522-z 36797275 PMC9935630

[54] Gao L Dong X Gong W Huang W Xue J Zhu Q. Acinar cell NLRP3 inflammasome and gasdermin D (GSDMD) activation mediates pyroptosis and systemic inflammation in acute pancreatitis. Br J Pharmacol. (2021) . 178:3533–52. doi: 10.1111/bph.15499 33871879

[55] Gao H Cao M Yao Y Hu W Sun H Zhang Y. Dysregulated microbiota-driven gasdermin D activation promotes colitis development by mediating IL-18 release. Front Immunol. (2021) . 12:750841. doi: 10.3389/fimmu.2021.750841 34721422 PMC8551709

[56] Wang J Yao J Liu Y Huang L. Targeting the gasdermin D as a strategy for ischemic stroke therapy. Biochem Pharmacol. (2021) . 188:114585. doi: 10.1016/j.bcp.2021.114585 33930348

[57] Jiang K Tu Z Chen K Xu Y Chen F Xu S. Gasdermin D inhibition confers antineutrophil-mediated cardioprotection in acute myocardial infarction. J Clin Invest. (2022) . 132:e151268. doi: 10.1172/JCI151268 34752417 PMC8718151

[58] Tanaka S Orita H Kataoka T Miyazaki M Saeki H Wada R. Gasdermin D represses inflammation-induced colon cancer development by regulating apoptosis. Carcinogenesis. (2023) . 44:341–9. doi: 10.1093/carcin/bgad005 36753047

[59] Van Laer L Huizing EH Verstreken M van Zuijlen D Wauters JG Bossuyt PJ. Nonsyndromic hearing impairment is associated with a mutation in DFNA5. Nat Genet. (1998) . 20:194–7. doi: 10.1038/2503 9771715

[60] Zhang Z Zhang Y Xia S Kong Q Li S Liu X. Gasdermin E suppresses tumour growth by activating anti-tumour immunity. Nature. (2020) . 579:415–20. doi: 10.1038/s41586-020-2071-9 PMC7123794 32188940

[61] Neel DV Basu H Gunner G Bergstresser MD Giadone RM Chung H. Gasdermin-E mediates mitochondrial damage in axons and neurodegeneration. Neuron. (2023) . 111:1222–1240.e9. doi: 10.1016/j.neuron.2023.02.019 36917977 PMC10121894

[62] Wei Y Lan B Zheng T Yang L Zhang X Cheng L. GSDME-mediated pyroptosis promotes the progression and associated inflammation of atherosclerosis. Nat Commun. (2023) . 14:929. doi: 10.1038/s41467-023-36614-w 36807553 PMC9938904

[63] Li Y Yuan Y Huang ZX Chen H Lan R Wang Z. GSDME-mediated pyroptosis promotes inflammation and fibrosis in obstructive nephropathy. Cell Death Differ. (2021) . 28:2333–50. doi: 10.1038/s41418-021-00755-6 PMC8329275 33664482

[64] Li W Sun J Zhou X Lu Y Cui W Miao L. Mini-review: GSDME-mediated pyroptosis in diabetic nephropathy. Front Pharmacol. (2021) . 12:780790. doi: 10.3389/fphar.2021.780790 34867412 PMC8637879

[65] Delmaghani S Del CFJ Michel V Leibovici M Aghaie A Ron U. Mutations in the gene encoding pejvakin, a newly identified protein of the afferent auditory pathway, cause DFNB59 auditory neuropathy. Nat Genet. (2006) . 38:770–8. doi: 10.1038/ng1829 16804542

[66] Mujtaba G Bukhari I Fatima A Naz S. A p.C343S missense mutation in PJVK causes progressive hearing loss. Gene. (2012) . 504:98–101. doi: 10.1016/j.gene.2012.05.013 22617256 PMC3534776

[67] Rathinam VA Fitzgerald KA. Inflammasome complexes: emerging mechanisms and effector functions. Cell. (2016) . 165:792–800. doi: 10.1016/j.cell.2016.03.046 27153493 PMC5503689

[68] Lamkanfi M Dixit VM. Mechanisms and functions of inflammasomes. Cell. (2014) . 157:1013–22. doi: 10.1016/j.cell.2014.04.007 24855941

[69] Chavarria-Smith J Vance RE. The NLRP1 inflammasomes. Immunol Rev. (2015) . 265:22–34. doi: 10.1111/imr.12283 25879281

[70] Huang Y Xu W Zhou R. NLRP3 inflammasome activation and cell death. Cell Mol Immunol. (2021) . 18:2114–27. doi: 10.1038/s41423-021-00740-6 PMC8429580 34321623

[71] Fu J Wu H. Structural mechanisms of NLRP3 inflammasome assembly and activation. Annu Rev Immunol. (2023) . 41:301–16. doi: 10.1146/annurev-immunol-081022-021207 PMC10159982 36750315

[72] Xu J Nunez G. The NLRP3 inflammasome: activation and regulation. Trends Biochem Sci. (2023) . 48:331–44. doi: 10.1016/j.tibs.2022.10.002 PMC10023278 36336552

[73] Li R Zhu S. NLRP6 inflammasome. Mol Aspects Med. (2020) . 76:100859. doi: 10.1016/j.mam.2020.100859 32386845

[74] Hara H Seregin SS Yang D Fukase K Chamaillard M Alnemri ES. The NLRP6 inflammasome recognizes lipoteichoic acid and regulates gram-positive pathogen infection. Cell. (2018) . 175:1651–1664.e14. doi: 10.1016/j.cell.2018.09.047 30392956 PMC6294477

[75] Levy M Thaiss CA Zeevi D Dohnalova L Zilberman-Schapira G Mahdi JA. Microbiota-modulated metabolites shape the intestinal microenvironment by regulating NLRP6 inflammasome signaling. Cell. (2015) . 163:1428–43. doi: 10.1016/j.cell.2015.10.048 PMC5665753 26638072

[76] Mullins B Chen J. NLRP9 in innate immunity and inflammation. Immunology. (2021) . 162:262–7. doi: 10.1111/imm.13290 PMC7884643 33283292

[77] Lugrin J Martinon F. The AIM2 inflammasome: Sensor of pathogens and cellular perturbations. Immunol Rev. (2018) . 281:99–114. doi: 10.1111/imr.12618 29247998

[78] Duncan JA Canna SW. The NLRC4 inflammasome. Immunol Rev. (2018) . 281:115–23. doi: 10.1111/imr.12607 PMC5897049 29247997

[79] Schnappauf O Chae JJ Kastner DL Aksentijevich I. The pyrin inflammasome in health and disease. Front Immunol. (2019) . 10:1745. doi: 10.3389/fimmu.2019.01745 31456795 PMC6698799

[80] Yang Y Wang H Kouadir M Song H Shi F. Recent advances in the mechanisms of NLRP3 inflammasome activation and its inhibitors. Cell Death Dis. (2019) . 10:128. doi: 10.1038/s41419-019-1413-8 30755589 PMC6372664

[81] Mangan MSJ Olhava EJ Roush WR Seidel HM Glick GD Latz E. Targeting the NLRP3 inflammasome in inflammatory diseases. Nat Rev Drug Discovery. (2018) . 17:588–606. doi: 10.1038/nrd.2018.97 30026524

[82] Miao EA Rajan JV Aderem A. Caspase-1-induced pyroptotic cell death. Immunol Rev. (2011) . 243:206–14. doi: 10.1111/j.1600-065X.2011.01044.x PMC3609431 21884178

[83] Liu X Zhang Z Ruan J Pan Y Magupalli VG Wu H. Inflammasome-activated gasdermin D causes pyroptosis by forming membrane pores. Nature. (2016) . 535:153–8. doi: 10.1038/nature18629 PMC5539988 27383986

[84] Shi J Zhao Y Wang Y Gao W Ding J Li P. Inflammatory caspases are innate immune receptors for intracellular LPS. Nature. (2014) . 514:187–92. doi: 10.1038/nature13683 25119034

[85] Yang D He Y Munoz-Planillo R Liu Q Nunez G. Caspase-11 requires the pannexin-1 channel and the purinergic P2X7 pore to mediate pyroptosis and endotoxic shock. Immunity. (2015) . 43:923–32. doi: 10.1016/j.immuni.2015.10.009 PMC4795157 26572062

[86] Deng W Bai Y Deng F Pan Y Mei S Zheng Z. Streptococcal pyrogenic exotoxin B cleaves GSDMA and triggers pyroptosis. Nature. (2022) . 602:496–502. doi: 10.1038/s41586-021-04384-4 35110732 PMC9703647

[87] LaRock DL Johnson AF Wilde S Sands JS Monteiro MP LaRock CN. Group A Streptococcus induces GSDMA-dependent pyroptosis in keratinocytes. Nature. (2022) . 605:527–31. doi: 10.1038/s41586-022-04717-x PMC9186297 35545676

[88] Li S Song J Liu J Zhou S Zhao G Li T. African swine fever virus infection regulates pyroptosis by cleaving gasdermin A (GSDMA) via active caspase-3 and caspase-4. J Biol Chem. (2024) . 300:107307. doi: 10.1016/j.jbc.2024.107307 38657868 PMC11163174

[89] Zhou Z He H Wang K Shi X Wang Y Su Y. Granzyme A from cytotoxic lymphocytes cleaves GSDMB to trigger pyroptosis in target cells. Science. (2020) 368:eaaz7548. doi: 10.1126/science.aaz7548 32299851

[90] Hansen JM de Jong MF Wu Q Zhang LS Heisler DB Alto LT. Pathogenic ubiquitination of GSDMB inhibits NK cell bactericidal functions. Cell. (2021) . 184:3178–3191.e18. doi: 10.1016/j.cell.2021.04.036 34022140 PMC8221529

[91] Feng Y Li M Yangzhong X Zhang X Zu A Hou Y. Pyroptosis in inflammation-related respiratory disease. J Physiol Biochem. (2022) . 78:721–37. doi: 10.1007/s13105-022-00909-1 PMC9684248 35819638

[92] Chen Q Shi P Wang Y Zou D Wu X Wang D. GSDMB promotes non-canonical pyroptosis by enhancing caspase-4 activity. J Mol Cell Biol. (2019) . 11:496–508. doi: 10.1093/jmcb/mjy056 30321352 PMC6734491

[93] Li X Zhang T Kang L Xin R Sun M Chen Q. Apoptotic caspase-7 activation inhibits non-canonical pyroptosis by GSDMB cleavage. Cell Death Differ. (2023) . 30:2120–34. doi: 10.1038/s41418-023-01211-3 PMC10482963 37591921

[94] Zhang JY Zhou B Sun RY Ai YL Cheng K Li FN. The metabolite alpha-KG induces GSDMC-dependent pyroptosis through death receptor 6-activated caspase-8. Cell Res. (2021) . 31:980–97. doi: 10.1038/s41422-021-00506-9 PMC8410789 34012073

[95] Tsuchiya K. Switching from apoptosis to pyroptosis: gasdermin-elicited inflammation and antitumor immunity. Int J Mol Sci. (2021) . 22:426. doi: 10.3390/ijms22010426 33406603 PMC7794676

[96] Burgener SS Leborgne NGF Snipas SJ Salvesen GS Bird PI Benarafa C. Cathepsin G inhibition by serpinb1 and serpinb6 prevents programmed necrosis in neutrophils and monocytes and reduces GSDMD-driven inflammation. Cell Rep. (2019) . 27:3646–3656.e5. doi: 10.1016/j.celrep.2019.05.065 31216481 PMC7350907

[97] Kambara H Liu F Zhang X Liu P Bajrami B Teng Y. Gasdermin D exerts anti-inflammatory effects by promoting neutrophil death. Cell Rep. (2018) . 22:2924–36. doi: 10.1016/j.celrep.2018.02.067 PMC5878047 29539421

[98] Sarhan J Liu BC Muendlein HI Li P Nilson R Tang AY. Caspase-8 induces cleavage of gasdermin D to elicit pyroptosis during Yersinia infection. Proc Natl Acad Sci U S A. (2018) . 115:E10888–97. doi: 10.1073/pnas.1809548115 PMC6243247 30381458

[99] Rogers C Fernandes-Alnemri T Mayes L Alnemri D Cingolani G Alnemri ES. Cleavage of DFNA5 by caspase-3 during apoptosis mediates progression to secondary necrotic/pyroptotic cell death. Nat Commun. (2017) . 8:14128. doi: 10.1038/ncomms14128 28045099 PMC5216131

[100] Jiang M Qi L Li L Li Y. The caspase-3/GSDME signal pathway as a switch between apoptosis and pyroptosis in cancer. Cell Death Discovery. (2020) . 6:112. doi: 10.1038/s41420-020-00349-0 33133646 PMC7595122

[101] Meng XM. Inflammatory mediators and renal fibrosis. Adv Exp Med Biol. (2019) . 1165:381–406. doi: 10.1007/978-981-13-8871-2_18 31399975

[102] Huang R Fu P Ma L. Kidney fibrosis: from mechanisms to therapeutic medicines. Signal Transduct Target Ther. (2023) . 8:129. doi: 10.1038/s41392-023-01379-7 36932062 PMC10023808

[103] Ruiz-Ortega M Rodrigues-Diez RR Lavoz C Rayego-Mateos S. Special issue “Diabetic nephropathy: diagnosis, prevention and treatment. J Clin Med. (2020) . 9:813. doi: 10.3390/jcm9030813 32192024 PMC7141346

[104] Kriz W Lowen J Grone HJ. The complex pathology of diabetic nephropathy in humans. Nephrol Dial Transplant. (2023) . 38:2109–19. doi: 10.1093/ndt/gfad052 PMC10539239 36918205

[105] Shao Y Deng S Tang W Huang L Xie Y Yuan S. Molecular mechanism of GSDMD mediated glomerular endothelial cells pyroptosis: An implying in the progression of diabetic nephropathy. Int Immunopharmacol. (2023) . 122:110632. doi: 10.1016/j.intimp.2023.110632 37451013

[106] An X Zhang Y Cao Y Chen J Qin H Yang L. Punicalagin protects diabetic nephropathy by inhibiting pyroptosis based on TXNIP/NLRP3 pathway. Nutrients. (2020) . 12:1516. doi: 10.3390/nu12051516 32456088 PMC7284711

[107] Cheng Q Pan J Zhou ZL Yin F Xie HY Chen PP. Caspase-11/4 and gasdermin D-mediated pyroptosis contributes to podocyte injury in mouse diabetic nephropathy. Acta Pharmacol Sin. (2021) . 42:954–63. doi: 10.1038/s41401-020-00525-z PMC8149386 32968210

[108] Cui X Li Y Yuan S Huang Y Chen X Han Y. Alpha-kinase1 promotes tubular injury and interstitial inflammation in diabetic nephropathy by canonical pyroptosis pathway. Biol Res. (2023) . 56:5. doi: 10.1186/s40659-023-00416-7 36732854 PMC9893546

[109] Li S Feng L Li G Liu R Ma C Wang L. GSDME-dependent pyroptosis signaling pathway in diabetic nephropathy. Cell Death Discovery. (2023) . 9:156. doi: 10.1038/s41420-023-01452-8 37169767 PMC10175547

[110] Wang Y Zhu X Yuan S Wen S Liu X Wang C. TLR4/NF-kappaB signaling induces GSDMD-related pyroptosis in tubular cells in diabetic kidney disease. Front Endocrinol (Lausanne). (2019) . 10:603. doi: 10.3389/fendo.2019.00603 31608008 PMC6761221

[111] Han Y Xu X Tang C Gao P Chen X Xiong X. Reactive oxygen species promote tubular injury in diabetic nephropathy: The role of the mitochondrial ros-txnip-nlrp3 biological axis. Redox Biol. (2018) . 16:32–46. doi: 10.1016/j.redox.2018.02.013 29475133 PMC5842313

[112] Hoi A Igel T Mok CC Arnaud L. Systemic lupus erythematosus. Lancet. (2024) . 403:2326–38. doi: 10.1016/S0140-6736(24)00398-2 38642569

[113] Anders HJ Saxena R Zhao MH Parodis I Salmon JE Mohan C. Lupus nephritis. Nat Rev Dis Primers. (2020) . 6:7. doi: 10.1038/s41572-019-0141-9 31974366

[114] Lech M Anders HJ. The pathogenesis of lupus nephritis. J Am Soc Nephrol. (2013) . 24:1357–66. doi: 10.1681/ASN.2013010026 PMC3752952 23929771

[115] Satoh M Reeves WH. Induction of lupus-associated autoantibodies in BALB/c mice by intraperitoneal injection of pristane. J Exp Med. (1994) . 180:2341–6. doi: 10.1084/jem.180.6.2341 PMC2191761 7964507

[116] Satoh M Kumar A Kanwar YS Reeves WH. Anti-nuclear antibody production and immune-complex glomerulonephritis in BALB/c mice treated with pristane. Proc Natl Acad Sci U S A. (1995) . 92:10934–8. doi: 10.1073/pnas.92.24.10934 PMC40545 7479913

[117] Zhang Z Song W Yan R. Gbp3 is associated with the progression of lupus nephritis by regulating cell proliferation, inflammation and pyroptosis. Autoimmunity. (2023) . 56:2250095. doi: 10.1080/08916934.2023.2250095 37621179

[118] Cao H Liang J Liu J He Y Ke Y Sun Y. Novel effects of combination therapy through inhibition of caspase-1/gasdermin D induced-pyroptosis in lupus nephritis. Front Immunol. (2021) . 12:720877. doi: 10.3389/fimmu.2021.720877 34867948 PMC8639704

[119] Luo G He Y Yang F Zhai Z Han J Xu W. Blocking GSDME-mediated pyroptosis in renal tubular epithelial cells alleviates disease activity in lupus mice. Cell Death Discovery. (2022) . 8:113. doi: 10.1038/s41420-022-00848-2 35279675 PMC8918340

[120] Norregaard R Mutsaers HAM Frokiaer J Kwon TH. Obstructive nephropathy and molecular pathophysiology of renal interstitial fibrosis. Physiol Rev. (2023) . 103:2827–72. doi: 10.1152/physrev.00027.2022 PMC10642920 37440209

[121] Wang K Liao Q Chen X. Research progress on the mechanism of renal interstitial fibrosis in obstructive nephropathy. Heliyon. (2023) . 9:e18723. doi: 10.1016/j.heliyon.2023.e18723 37593609 PMC10428074

[122] Wang Y Li Y Chen Z Yuan Y Su Q Ye K. GSDMD-dependent neutrophil extracellular traps promote macrophage-to-myofibroblast transition and renal fibrosis in obstructive nephropathy. Cell Death Dis. (2022) . 13:693. doi: 10.1038/s41419-022-05138-4 35941120 PMC9360039

[123] Wu M Xia W Jin Q Zhou A Wang Q Li S. Gasdermin E deletion attenuates ureteral obstruction- and 5/6 nephrectomy-induced renal fibrosis and kidney dysfunction. Front Cell Dev Biol. (2021) . 9:754134. doi: 10.3389/fcell.2021.754134 34746148 PMC8567074

[124] Zhang Y Zhang R Han X. Disulfiram inhibits inflammation and fibrosis in a rat unilateral ureteral obstruction model by inhibiting gasdermin D cleavage and pyroptosis. Inflammation Res. (2021) . 70:543–52. doi: 10.1007/s00011-021-01457-y 33851234

[125] Jia Q Zhang X Hao G Zhao Y Lowe S Han L. Tongluo yishen decoction ameliorates renal fibrosis via NLRP3-mediated pyroptosis *in vivo* and *in vitro* . Front Pharmacol. (2022) . 13:936853. doi: 10.3389/fphar.2022.936853 35873572 PMC9298980

[126] Feng M Luo F Wu H Chen Y Zuo J Weng X. Network pharmacology analysis and machine-learning models confirmed the ability of yiShen huoXue decoction to alleviate renal fibrosis by inhibiting pyroptosis. Drug Des Devel Ther. (2023) . 17:3169–92. doi: 10.2147/DDDT.S420135 PMC10612518 37900883

[127] Perazella MA Herlitz LC. The crystalline nephropathies. Kidney Int Rep. (2021) . 6:2942–57. doi: 10.1016/j.ekir.2021.09.003 PMC8640557 34901567

[128] Piani F Sasai F Bjornstad P Borghi C Yoshimura A Sanchez-Lozada LG. Hyperuricemia and chronic kidney disease: to treat or not to treat. J Bras Nefrol. (2021) . 43:572–9. doi: 10.1590/2175-8239-JBN-2020-U002 PMC8940113 33704350

[129] Ermer T Eckardt KU Aronson PS Knauf F. Oxalate, inflammasome, and progression of kidney disease. Curr Opin Nephrol Hypertens. (2016) . 25:363–71. doi: 10.1097/MNH.0000000000000229 PMC4891250 27191349

[130] Llanos M Kwon A Herlitz L Shafi T Cohen S Gebreselassie SK. The clinical and pathological characteristics of patients with oxalate nephropathy. Kidney360. (2024) . 5:65–72. doi: 10.34067/KID.0000000000000340 38095544 PMC10833593

[131] Gan XG Wang ZH Xu HT. Mechanism of miRNA-141-3p in Calcium Oxalate-Induced Renal Tubular Epithelial Cell Injury via NLRP3-Mediated Pyroptosis. Kidney Blood Press Res. (2022) . 47:300–8. doi: 10.1159/000521795 35081536

[132] Chen Y Yang S Kong H Wang Q Chen S Wang X. Oxalate−induced renal pyroptotic injury and crystal formation mediated by NLRP3−GSDMD signaling *in vitro* and *in vivo* . Mol Med Rep. (2023) . 28:209. doi: 10.3892/mmr.2023.13096 37732544 PMC10540023

[133] Liu J Yang K Jin Y Liu Y Chen Y Zhang X. H3 relaxin protects against calcium oxalate crystal-induced renal inflammatory pyroptosis. Cell Prolif. (2020) . 53:e12902. doi: 10.1111/cpr.12902 32945585 PMC7574868

[134] Yao K Zhang ZH Liu MD Niu FW Li X Ding DM. Melatonin alleviates intrarenal CaOx crystals deposition through inhibiting LPS-induced non-canonical inflammasome-mediated renal tubular epithelial cells pyroptosis. Int Immunopharmacol. (2023) 124:110796. doi: 10.1016/j.intimp.2023.110796 37633237

[135] Pan J Zhang C Shi M Guo F Liu J Li L. Ethanol extract of Liriodendron chinense (Hemsl.) Sarg barks attenuates hyperuricemic nephropathy by inhibiting renal fibrosis and inflammation in mice. J Ethnopharmacol. (2021) . 264:113278. doi: 10.1016/j.jep.2020.113278 32841699

[136] Yang L Wang B Ma L Fu P. Traditional Chinese herbs and natural products in hyperuricemia-induced chronic kidney disease. Front Pharmacol. (2022) . 13:971032. doi: 10.3389/fphar.2022.971032 36016570 PMC9395578

[137] Johnson RJ. Intestinal hyperuricemia as a driving mechanism for CKD. Am J Kidney Dis. (2023) . 81:127–30. doi: 10.1053/j.ajkd.2022.08.001 36167757

[138] Ma L Shen R Jiao J Lin X Zhai B Xu A. Gasdermin D promotes hyperuricemia-induced renal tubular injury through RIG-I/caspase-1 pathway. iScience. (2023) . 26:108463. doi: 10.1016/j.isci.2023.108463 38187191 PMC10767184

[139] Hu Y Shi Y Chen H Tao M Zhou X Li J. Blockade of autophagy prevents the progression of hyperuricemic nephropathy through inhibiting NLRP3 inflammasome-mediated pyroptosis. Front Immunol. (2022) . 13:858494. doi: 10.3389/fimmu.2022.858494 35309342 PMC8924517

[140] Ding Z Zhao J Wang X Li W Chen C Yong C. Total extract of Abelmoschus manihot L. alleviates uric acid-induced renal tubular epithelial injury via inhibition of caspase-8/caspase-3/NLRP3/GSDME signaling. Front Pharmacol. (2022) . 13:907980. doi: 10.3389/fphar.2022.907980 36052125 PMC9424722

[141] Kujal P Vernerova Z. 5/6 nephrectomy as an experimental model of chronic renal failure and adaptation to reduced nephron number. Cesk Fysiol. (2008) . 57:104–9. 19526664

[142] Adam RJ Williams AC Kriegel AJ. Comparison of the surgical resection and infarct 5/6 nephrectomy rat models of chronic kidney disease. Am J Physiol Renal Physiol. (2022) 322:F639–54. doi: 10.1152/ajprenal.00398.2021 PMC9076416 35379002

[143] Tian X Zeng Y Tu Q Jiao Y Yao S Chen Y. Butyrate alleviates renal fibrosis in CKD by regulating NLRP3-mediated pyroptosis via the STING/NF-kappaB/p65 pathway. Int Immunopharmacol. (2023) 124:111010. doi: 10.1016/j.intimp.2023.111010 37852118

